# Molecular Ecology of Isoprene-Degrading Bacteria

**DOI:** 10.3390/microorganisms8070967

**Published:** 2020-06-27

**Authors:** Ornella Carrión, Terry J. McGenity, J. Colin Murrell

**Affiliations:** 1 School of Environmental Sciences, Norwich Research Park, University of East Anglia, Norwich NR4 7TJ, UK; 2 School of Life Sciences, University of Essex, Colchester CO4 3SQ, UK; tjmcgen@essex.ac.uk

**Keywords:** climate, BVOC, isoprene, isoprene monooxygenase, *isoA*, DNA stable-isotope probing

## Abstract

Isoprene is a highly abundant biogenic volatile organic compound (BVOC) that is emitted to the atmosphere in amounts approximating to those of methane. The effects that isoprene has on Earth’s climate are both significant and complex, however, unlike methane, very little is known about the biological degradation of this environmentally important trace gas. Here, we review the mechanisms by which bacteria catabolise isoprene, what is known about the diversity of isoprene degraders in the environment, and the molecular tools currently available to study their ecology. Specifically, we focus on the use of probes based on the gene encoding the α-subunit of isoprene monooxygenase, *isoA*, and DNA stable-isotope probing (DNA-SIP) alone or in combination with other cultivation-independent techniques to determine the abundance, diversity, and activity of isoprene degraders in the environment. These parameters are essential in order to evaluate how microbes might mitigate the effects of this important but neglected climate-active gas. We also suggest key aspects of isoprene metabolism that require further investigation in order to better understand the global isoprene biogeochemical cycle.

## 1. Isoprene and Climate

Isoprene (2-methyl-1,3-butadiene), with global annual emissions to the atmosphere of approximately 500 Tg, is the most abundantly produced biogenic volatile organic compound (BVOC) on Earth. This is similar in magnitude to the emissions of all other BVOCs combined and equal to global emissions of methane per year [1,2]. Due to its abundance, volatility (boiling point of 34 °C), and high reactivity (due to the presence of two C=C bonds) isoprene plays a significant and complex role in atmospheric chemistry, and hence, climate [3]. In rural environments with low levels of nitrogen oxides (NOx), isoprene reacts with hydroxyl radicals (OH), reducing the oxidising capacity of the atmosphere [1]. This in turn increases the residence time of greenhouse gases, such as methane, and thus enhances global warming [4,5]. In urban environments, nitric oxide (NO) is typically present at high concentrations and reacts with isoprene to form nitrogen dioxide (NO_2_). The photolysis of NO_2_ increases the tropospheric levels of ozone [3], a greenhouse gas which has detrimental effects on air quality and plant and animal health [6]. Conversely, the atmospheric oxidation of isoprene can also form secondary aerosols that act as cloud condensation nuclei, resulting in a global cooling effect [7,8].

## 2. Global Isoprene Emissions

The vast majority of isoprene (~90%) emitted globally is produced by terrestrial plants [9]. However, there is a lot of variation in isoprene production, particularly in trees, which are major sources. For example, not all trees produce isoprene, and high and low emitters can be found even among closely related species [10,11,12]. In isoprene-emitting plants, isoprene is synthesised in the chloroplast from dimethylallyl diphosphate (DMAPP) in a reaction catalysed by isoprene synthase [13]. Although typically ~2% of carbon photosynthetically fixed in high isoprene-emitting trees can be diverted to the production of isoprene, and in some cases even more [14], it is striking that its role in plants is not yet fully understood. Previous studies have reported that isoprene can confer protection against thermal and oxidative stress (reviewed in [15]). However, the molecular mechanisms behind these processes have not yet been completely elucidated. Other proposed roles for isoprene include effects on plant growth, insect herbivory and, more recently, the regulation of gene expression, as well as effects on the proteome and the metabolome of plants (reviewed in [15]). 

Since trees are the major source of isoprene in the biosphere, the increase in land usage dedicated to the cultivation of high isoprene-emitting trees (e.g., poplar (*Populus* spp.), willow (*Salix* spp.), eucalyptus (*Eucalyptus* spp.), and oil palm (*Elaeis guineensis*)), especially for biofuel and food production, has raised serious concerns about the impact on air quality and human health [6,16,17].

The remaining 10% of isoprene emissions to the atmosphere is attributed to bacteria, fungi, algae, and animals [18,19,20,21,22]. Bacteria reported to synthesise isoprene to date include strains from the *Proteobacteria, Actinobacteria*, and *Firmicutes* phyla, with *Bacillus* being the best-characterised isoprene-producing genus [21,23,24,25]. However, the biosynthetic pathways for isoprene in bacteria and indeed why isoprene is produced by microbes is uncertain (reviewed in [26]).

In the marine environment, isoprene emissions are estimated to range from 0.1 to 11.6 Tg per year depending on the methodology used (reviewed in [26]) and mainly originate from phytoplankton, seaweeds, and heterotrophic bacteria [20,27,28]. The role of isoprene in marine environments is not well understood. It has been suggested that it might confer protection against high temperatures, light intensity, and oxidative stress [27,29,30,31]. Other environments that have been reported to emit isoprene, but remain relatively unexplored, include wetlands and freshwater lakes [32,33]. One further source of isoprene to the atmosphere is anthropogenic emissions of isoprene (estimated at ~0.8 Tg per year) [34]. These are due to the production of isoprene for use as a bulk chemical to synthesise a wide range of industrial products, including synthetic rubber, elastomers and pharmaceuticals, vehicle exhausts, and biomass burning [34,35,36]. 

## 3. Biological Sinks for Isoprene

It has been known for over 20 years that soils act as a biological sink for isoprene at environmentally relevant concentrations due to field chamber and continuous-flow experiments performed with temperate, tropical, and boreal soils [37,38,39,40]. In the marine environment, Acuña Alvarez et al. [41] showed the capacity for biological degradation of isoprene in estuarine, coastal, and open marine waters and sediments. They also demonstrated that isoprene-degrading bacteria isolated from these environments consumed isoprene naturally produced by microalgal cultures. More recently, experiments conducted with phyllosphere samples from high isoprene-emitting trees from temperate and tropical regions (poplar, willow, and oil palm trees) have shown that this environment is likely to be an important source of isoprene-degrading microorganisms [42,43,44,45]. However, despite these findings, the significance of the biological consumption of isoprene is still a key aspect of the global isoprene cycle that needs to be addressed. 

## 4. Diversity of Isoprene-degrading Bacteria

Early soil enrichments with isoprene led to the isolation of bacteria that could use this trace gas as a sole carbon and energy source. These microorganisms were tentatively assigned to the genera *Rhodococcus, Nocardia, Arthrobacter*, and *Alcaligenes* [37,46,47,48]. Studies with rubber-contaminated soil yielded *Pseudomonas, Klebsiella*, and *Alcaligenes* isoprene-degrading strains [49]. Isoprene degraders of the genera *Arthrobacter*, *Bacillus*, *Pseudomonas, Sphingobacterium, Sphingobium*, and *Pantoea* have been also isolated from soils and leaves from tropical teak (*Tectona grandis*) and mahua trees (*Madhuca latifolia*) [50]. However, none of these strains was characterised in detail. Isoprene enrichments with garden and tyre dump soils, as well as soils in the vicinity of willow trees, have also led to the isolation of several *Rhodococcus* species and novel *Nocardioides, Ramlibacter*, and *Variovorax* strains, which degrade isoprene [51,52,53,54,55,56]. Recent studies exploring the phyllosphere of poplar, willow, and oil palm trees have shown that this environment harbours a considerable variety of isoprene-degrading isolates, including members of the genera *Rhodococcus, Gordonia, Sphingopyxis*, and *Variovorax* [42,51,52,55]. Moreover, Acuña Alvarez et al. [41] reported the isolation, from marine and estuarine environments, of several *Actinobacteria* (*Leifsonia, Gordonia, Mycobacterium, Rhodococcus*) and *Alphaproteobacteria* (*Stappia, Loktanella, Shinella*) that could grow on isoprene, with *Gordonia* and *Mycobacterium* being the most well characterised [57]. Thus far, freshwater environments have not been explored in detail, however, van Hylckama Vlieg et al. [58] isolated *Rhodococcus* sp. AD45 from freshwater sediments, which has become a key bacterial strain for the study of isoprene metabolism [43,58,59,60,61]. Details about the isoprene-degrading strains belonging to these genera are provided in [62]. Finally, to our knowledge, no anaerobic bacteria, archaea, or fungi have been reported to use isoprene as a sole carbon and energy source, although Kronen et al. [63] have recently shown that strict anaerobes can use isoprene as an electron acceptor to support homoacetogenesis, and thus anaerobic degradation of isoprene also warrants further attention.

## 5. Isoprene Degradation Pathway

**Figure 1:**
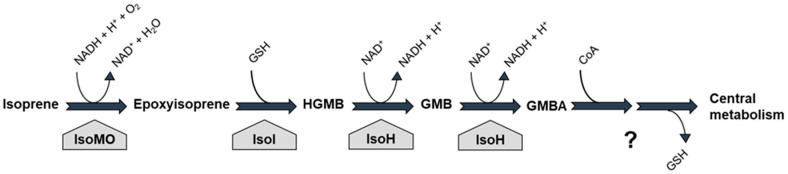
Isoprene degradation pathway in the model isoprene-utilising bacterium *Rhodococcus* sp. AD45. Enzymes: IsoMO, isoprene monooxygenase; IsoI, glutathione-*S*-transferase; IsoH, dehydrogenase; HGMB, 1-hydroxy-2-glutathionyl-2-methyl-3-butene; GMB, 2-glutathionyl-2-methyl-3-butenal; GMBA, 2-glutathionyl-2-methyl-3-butenoic acid; GSH, reduced glutathione. The question mark indicates uncertainty in the details of the catabolic pathway from GMBA.

In the best-characterised isoprene-degrading bacterium, *Rhodococcus* sp. AD45, isoprene is first oxidised to epoxyisoprene in a reaction catalysed by isoprene monooxygenase (IsoMO; Figure 1), a member of the soluble diiron monooxygenase (SDIMO) family [64]. Then, a glutathione-*S*-transferase (IsoI) conjugates the reactive epoxide with glutathione, forming 1-hydroxy-2-glutathionyl-2-methyl-3-butene (HGMB), which is further converted to glutathionyl-2-methyl-3-butenoate (GMBA) by a dehydrogenase (IsoH; [58,59]; Figure 1). However, the downstream catabolic pathway has not yet been elucidated. It is assumed that, after the removal of glutathione and β-oxidation, GMBA intermediates enter central metabolism, thus allowing *Rhodococcus* sp. AD45 to grow on isoprene as a sole carbon and energy source. 

**Figure 2:**
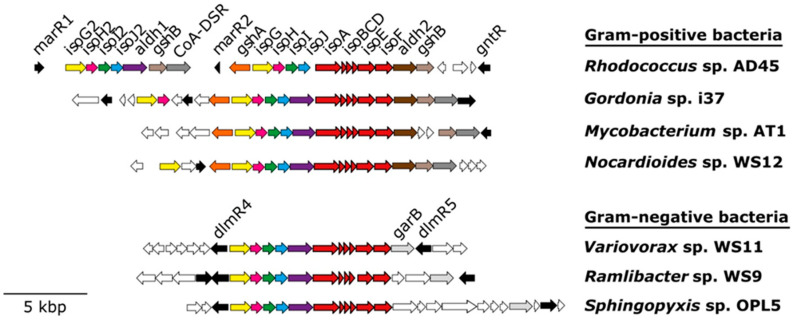
Isoprene degradation gene clusters from representative Gram-positive and Gram-negative isoprene-degrading bacteria. Genes encoding IsoMO (*isoABCDEF*) are coloured in red. Regulatory genes are shown in black. Adjacent genes not suspected to be involved in isoprene degradation are coloured in white.

All isoprene degraders characterised to date contain six genes (*isoABCDEF*) encoding the α_2_β_2_γ_2_ oxygenase (IsoABE), reductase (IsoF), Rieske-type ferredoxin (IsoC), and coupling protein (IsoD) that form IsoMO. Four additional genes (*isoGHIJ*), which encode a putative coenzyme A transferase, a dehydrogenase, and two glutathione transferases [58,59,60], are located immediately upstream (5’) of the structural genes *isoABCDEF*, which appears to be a typical layout for *iso* genes in all bona fide isoprene-degrading bacteria studied in detail to date (Figure 2). It is interesting that in some isoprene degraders, such as *Rhodococcus* sp. AD45 and *Variovorax* sp. WS11, the isoprene degradation gene cluster is encoded on a megaplasmid [53,61], suggesting that these bacteria might have acquired the ability to grow on isoprene by horizontal gene transfer.

Accessory genes invariably found within the isoprene degradation gene clusters of Gram-positive bacteria include: *aldH* (encoding an aldehyde dehydrogenase), *gshA* (glutamate cysteine ligase), *gshB* (glutathione synthetase), and *CoA-DSR* (CoA-disulfide reductase) [61]. Three putative transcriptional regulators (*marR1, marR2* and *gntR*) are also located within the isoprene degradation gene cluster of *Rhodococcus* sp. AD45 [61]. However, in Gram-negative isoprene-degrading strains, such as *Variovorax* sp. WS11, *aldH* and *garB* (encoding a glutathione disulfide reductase) are present within the isoprene degradation gene cluster, whereas genes involved in the biosynthesis of glutathione (*gshAB*) are located on the chromosome [53] (Figure 2). Finally, two putative LysR-type transcriptional regulators (*dmlR4* and *dmlR5*) have been identified flanking the isoprene degradation gene cluster of *Variovorax* sp. WS11 [53]. 

Thus, there are still some uncertainties within the isoprene degradation pathway, such as the downstream catabolic steps from GMBA, the role of the accessory genes, and the exact mechanisms by which isoprene degradation is regulated, although it has been shown that epoxyisoprene upregulates the expression of *iso* genes at a higher level than isoprene itself in *Rhodococcus* sp. AD45 and *Variovorax* sp. WS11 [53,61]. Targeted mutagenesis and expression studies, along with the construction of reporter strains, should be a fruitful approach to elucidate the full isoprene degradation pathway and to decipher the nature and mechanisms of action of putative promoters, transcriptional activators, and inducers, all of which are currently ongoing in our laboratory.

## 6. Molecular Techniques to Study the Ecology of Isoprene Degraders

### 6.1. isoA Probes

As described above, isoprene degradation seems to be a widespread trait across many phylogenetically diverse bacteria. Therefore, the use of 16S rRNA primers alone to identify isoprene degraders in environmental samples would not be successful. However, molecular ecology studies targeting genes encoding key subunits of other SDIMOs, such as *mmoX* (soluble methane monooxygenase; sMMO), have greatly enhanced our knowledge of the diversity and abundance of methane-oxidising bacteria in many environments [65,66,67,68]. The availability of genes essential for isoprene metabolism has enabled us to follow a similar approach. The *isoA* gene (a homolog of *mmoX*), encodes the α-subunit of the IsoMO which contains the diiron centre at the putative active site of SDIMO enzymes [64]. This *isoA* gene is conserved in all characterised isoprene degraders and can be readily distinguished from the α-subunits from other SDIMOs [51,69]. Therefore, *isoA* constituted an obvious target for the design of probes to study isoprene degraders in environmental samples. Following these principles, El Khawand et al. [51] aligned *isoA* sequences from bona fide isoprene-degrading bacteria and designed a PCR primer set targeting this gene. This *isoA* primer set yielded a PCR product with DNA extracted from a wide range of isoprene degraders and environmental samples, including leaves, soils, and estuarine and marine water and sediments, but no PCR product was observed with DNA from non-isoprene-degrading strains that harboured other SDIMO enzymes [51]. In addition, the IsoA sequences retrieved from environmental samples showed >86% amino acid identity to IsoA from ratified isoprene degraders, which at the time were predominantly *Actinobacteria*, and could be clearly separated into two distinct groups: those retrieved from terrestrial and estuary samples of lower salinity and those recovered from marine environments, including the mouth of the estuary [51]. Therefore, this approach confirmed *isoA* as an excellent marker gene for isoprene degradation and contributed to the expansion of the known diversity of isoprene degraders in terrestrial, estuarine, and marine environments [57]. 

**Figure 3:**
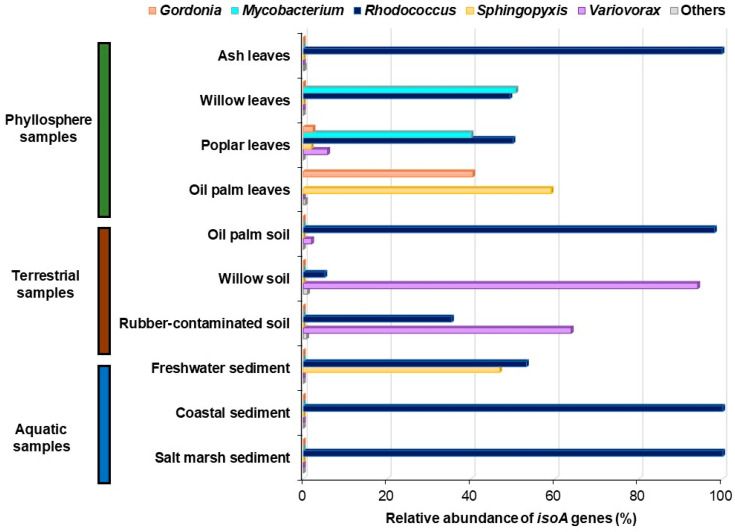
Relative abundance and diversity of *isoA* genes in environmental samples enriched with isoprene analysed by amplicon sequencing. Amplicon sequence variants (ASVs) closely related to IsoA from *Gordonia* are shown in orange; ASVs with the highest homology to IsoA from *Mycobacterium* are represented in light blue; ASVs encoding proteins homologous to *Rhodococcus* IsoA are shown in dark blue; ASVs with the highest homology to *Sphingopyxis* IsoA are coloured in yellow; ASVs closely related to IsoA from *Variovorax* are represented in purple. Data from [69].

However, the isolation and characterisation of novel isoprene-degrading *Proteobacteria*, such as *Variovorax, Sphingopyxis*, or *Ramlibacter* [56], revealed a higher diversity of *isoA* sequences than previously thought and emphasised the need to refine the *isoA* probes to cover all the isoprene degraders characterised to date. To that end, Carrión et al. [69] aligned 38 *isoA* genes from confirmed isoprene degraders, along with 18 sequences from metagenomes obtained from environmental samples enriched with isoprene that had ≥ 98% coverage and ≥ 85% amino acid identity to ratified IsoA sequences. Sixteen genes encoding α-subunits from other groups of SDIMOs were also included in the alignment to guide the specific amplification of *isoA*. After identifying the conserved regions within *isoA*, a primer set that spans the first iron centre of the IsoMO α-subunit was designed [69]. This new primer set yielded a specific PCR product of 497 bp with DNA from all 30 positive control isoprene-degrading strains tested, but no amplification products were obtained with DNA from the 12 non-isoprene-degrading strains used in this study as negative controls. To further validate the specificity of the new *isoA* probes, clone libraries from phyllosphere, soils, freshwater, and marine samples enriched with isoprene were made. Sequences retrieved by PCR with DNA from these environmental samples showed ≥ 84% amino acid identity to IsoA from ratified isoprene degraders but were ≤ 70% identical to α-subunits from other closely related SDIMOs, confirming that these *isoA* probes are a valuable tool to study the ecology of isoprene degraders [69]. Subsequently, Carrión et al. [69] used the new *isoA* primer set to further investigate the diversity of *isoA* genes in a wider range of environmental samples enriched with isoprene by *isoA* amplicon sequencing. The sequencing data revealed that phyllosphere samples tested harboured the highest diversity of *isoA* genes, with sequences homologous to IsoA from *Rhodococcus, Gordonia, Mycobacterium, Sphingopyxis*, and *Variovorax* (Figure 3). Soils collected from beneath high isoprene-emitting trees, such as oil palm and willow, and also rubber-contaminated soil, were dominated by *isoA* genes closely related to IsoA from *Rhodoccoccus* and *Variovorax*. The most abundant sequences from freshwater sediment were similar to *Rhodococcus* and *Sphingopyxis* IsoA, whereas coastal and marine sediments were overwhelmingly dominated by sequences closely related to IsoA from *Rhodoccoccus* (Figure 3). However, it is worth noting that the phylogeny of these *isoA* sequences might need to be reassessed when new genera of isoprene degraders are isolated, since the number of fully characterised isoprene-degrading strains to date is still limited. The existence of novel isoprene degraders with diverse *isoA* genes in the environments studied by Carrión et al. [69] is supported by the fact that some sequences recovered in this study, especially those from freshwater sediment, showed only 83–91% amino acid identity to IsoA from confirmed isoprene-degrading bacteria [69]. This information provided by the new *isoA* probes can be used now to design targeted isolation strategies for novel isoprene-utilising strains. The characterisation of new genera of isoprene degraders will contribute to a more robust IsoA database, which in turn will enable a more accurate phylogenetic assessment of the *isoA* genes retrieved from environmental samples, and thus a more precise picture of the main players in isoprene degradation in a particular habitat. 

**Figure 4:**
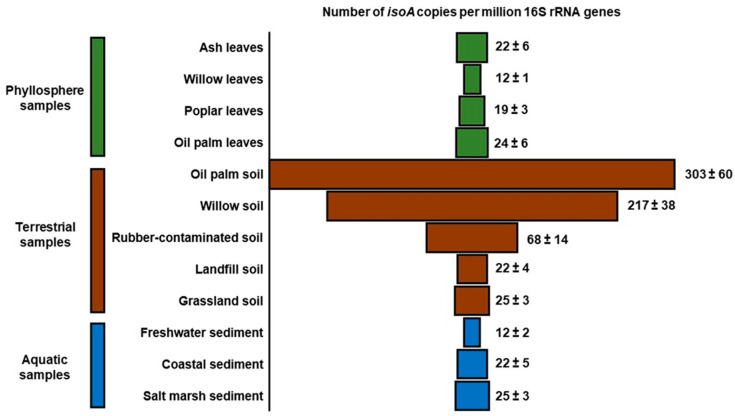
Relative abundance of isoprene degraders in natural (non-enriched) environmental samples determined by qPCR. Copies of *isoA* are normalised to the 16S rRNA gene copy number in each sample. Phyllosphere samples are coloured in green, soils in brown, and aquatic environments in blue. Data from [69].

The new *isoA* primer set has also shown to be a key tool to study the distribution and abundance of isoprene degraders in natural (non-enriched) environmental samples. In qPCR assays using the *isoA* probes, it was revealed that isoprene degraders are widespread in the environment [69] (Figure 4). Nevertheless, soils in the vicinity of high isoprene-emitting trees, such as oil palm and willow, contained the highest numbers of *isoA* genes when normalised to the abundance of 16S rRNA genes (~200 to 300 *isoA* genes per million copies of 16S rRNA genes) [69]. This value was approximately 10-fold higher than the relative abundance of *isoA* observed in leaves taken from the same trees, indicating that soils might be a more important sink for isoprene than previously thought. Another interesting observation from this study was that the numbers of *isoA* genes in the phyllosphere of high isoprene-emitting trees were similar to those found in leaves from an ash tree (*Fraxinus* spp.), a low isoprene emitter [69,70]. However, a larger number of samples are required to confirm these results statistically. Finally, although several isoprene-degrading strains have been isolated from freshwater and marine samples [41,57,58], it was surprising that these environments contained similar numbers of *isoA* sequences per million copies of 16S rRNA genes to those found in the phyllosphere samples tested, since the leaves of trees are considered to be the main source of isoprene globally [69] (Figure 4). These preliminary results are from a very small sample of different environments and there is a considerable need to study in more detail isoprene production and consumption in terrestrial, freshwater, and marine environments. Therefore, estimations of isoprene production and consumption rates, coupled with surveys of *isoA* genes (diversity, quantity, and activity), will enable researchers to obtain a better understanding of the role of microbes in the global isoprene biogeochemical cycle. 

These *isoA* probes are a relevant and successful tool to study the ecology of isoprene degraders, however, it is important to continuously refine them based on new information, and combine them with other cultivation-independent techniques, such as DNA stable-isotope probing (DNA-SIP), to enhance the detection of uncultivated isoprene-degrading bacteria with more diverse *isoA* genes or with novel isoprene catabolic pathways.

### 6.2. DNA Stable-Isotope Probing (DNA-SIP)

DNA-SIP can be a powerful technique to link the identity and function of microbes in the environment [65,71]. It relies on the incubation of environmental samples with ^13^C-labelled isoprene for a sufficient time for the active isoprene-degrading community to assimilate enough ^13^C-isoprene into their biomass to allow the detection and analysis of ^13^C-labelled DNA. The ^13^C-labelled DNA from the active isoprene degraders can be separated from the unlabelled DNA from the rest of the microbial community (the non-isoprene-consuming population) by isopycnic ultracentrifugation. Once separated, the ^13^C-labelled DNA can be analysed using a variety of techniques, such as amplicon sequencing or metagenomics [72]. 

Several DNA-SIP experiments with ^13^C-labelled isoprene have been conducted with samples from terrestrial, phyllosphere, and marine environments [42,43,45,51,56,57]. El Khawand et al. [51] and Larke-Mejía et al. [56] investigated the composition of the active isoprene-degrading community from soil beneath willow trees using DNA-SIP with high and low concentrations of isoprene (Table 1). Although both studies revealed *Rhodococcus* and members of the *Comamonadaceae* family (*Comamonas, Ramlibacter*, and *Variovorax*) as the main isoprene degraders in soils surrounding willow trees, *Rhodococcus* dominated the isoprene-utilising bacterial community at high concentrations of isoprene, whereas *Comamonadaceae* were more abundant when low concentrations were used (Table 1). In addition, the metagenomic sequencing of the ^13^C-labelled DNA allowed the identification of isoprene degradation gene clusters closely related to those found in *Rhodococcus, Nocardioides, Ramlibacter*, and *Sphingopyxis* [56]. This information was subsequently used to isolate representative isoprene-degrading strains from these genera [54,56], which in turn have significantly expanded the existing database of isoprene degradation genes and paved the way for the design of the new *isoA* probes. Recent studies have also shown that soils in the vicinity of oil palm trees harbour a very diverse isoprene-utilising bacterial community, with *Aquabacterium, Novosphingobium, Pelomonas, Rhodoblastus, Saccharibacter, Sphingomonas*, and *Rhodococcus* being the main genera enriched in samples incubated with ^13^C-isoprene [42,45,56] (Table 1). However, no isolates from these genera were obtained in these studies and the pathway that these ^13^C-labelled bacteria might use to catabolise isoprene has not yet been elucidated, except for *Novosphingobium*, for which a metagenome-assembled genome (MAG) containing a full isoprene degradation gene cluster with 76.2–100% identity at the derived amino acid level with the corresponding polypeptides from *Sphingopyxis* sp. OPL5 was recovered [42]. 

In addition to soils, DNA-SIP has also been used to identify active isoprene degraders in the phyllosphere. DNA-SIP experiments performed with leaf washings from white poplar showed that *Rhodococcus* and *Variovorax* played a major role in isoprene degradation in this environment (Table 1). Transcriptomic analyses also revealed the presence of RNA encoding *iso* genes of both genera in microcosms set up with the same phyllosphere samples incubated with ^13^C-isoprene [43]. Moreover, *Rhodococcus, Pseudonocardia*, and *Variovorax* MAGs containing genes involved in isoprene metabolism were reconstructed from the metagenomic sequencing of the ^13^C-labelled DNA. The *Rhodococcus* MAG was the most abundant bin recovered and was phylogenetically close to *Rhodococcus* sp. ACPA4 (98.7% average nucleotide identity), an isoprene degrader isolated from poplar leaves [52]. The *Pseudonocardia* MAG contained six genes homologous to those involved in isoprene degradation, including *isoA*, with 75–93% identity at the derived amino acid level to the corresponding proteins from *Rhodococcus* sp. AD45 [43], which is interesting because there are no known isoprene degraders from this genus. However, the isolation of representative strains from *Pseudonocardia* spp. is needed to confirm their ability to consume isoprene. Conversely, the *Variovorax* MAG contained a complete isoprene degradation gene cluster in an identical layout to those of many ratified isoprene-utilising isolates [43] but had a low amino acid identity (42–71%) to the homologous Iso enzymes from *Rhodococcus* sp. AD45. The IsoMO from the *Variovorax* MAG was subsequently shown to oxidise isoprene when cloned and expressed in a non-isoprene-degrading *Rhodococcus* strain, confirming that it is a functional enzyme, and illustrating how the heterologous expression of putative *iso* gene clusters can be a good way to test for activity [43]. These types of DNA-SIP experiments have guided the targeted isolation of isoprene-degrading *Variovorax* strains and have provided a new “workhorse” Gram-negative strain, *Variovorax* sp. WS11, which is currently being characterised in detail [53,56], and is being compared to the Gram-positive *Rhodococcus* sp. AD45. In addition to poplar leaves, the phyllosphere of oil palm, one of the most prolific isoprene-producing trees, has also been explored using DNA-SIP. These experiments have thus far revealed that the main players in isoprene degradation from this environment appear to be members of the genera *Aquincola, Gordonia, Sphingomonas*, and *Zoogloea* [42,45] (Table 1).

Finally, in the marine environment, Johnston et al. [57] used DNA-SIP to study isoprene degradation in surface estuarine sediments and showed that the active isoprene-utilising bacteria in this environment were mainly *Actinobacteria*, including *Mycobacterium, Microbacterium, Gordonia*, and *Rhodococcus* (Table 1). However, no members of *Proteobacteria* were highly enriched in the ^13^C-labelled microbial community [57].

Table 1 summarises the main cultivation-independent studies focusing on isoprene degradation, highlighting that, although *Rhodococcus* is ecologically flexible and widespread across terrestrial, phyllosphere, and estuarine samples, each environment that has been examined so far has its own specific isoprene-degrading community. Moreover, the limited studies carried out to date suggest that genera other than *Rhodococcus* dominate the isoprene-utilising bacterial community when lower concentrations of isoprene are added (Table 1). Thus, further cultivation-independent studies exploring contrasting environments are required to expand our understanding of the diversity of isoprene degraders and, consequently, to better assess their contribution to the isoprene biogeochemical cycle. Coupling these studies with cultivation, pathway analysis, and probe refinement will further improve our understanding of isoprene degradation.

## 7. Outlook

Despite isoprene being released to the atmosphere in large amounts every year and having major impacts on the climate, we are only starting to understand its biogeochemical cycle, and there are several questions that need to be addressed. Firstly, more accurate estimations of isoprene production in the biosphere are required, especially in marine environments, in order to improve global models of isoprene fluxes and to predict how fluxes are going to be affected by climate change. Secondly, although the isoprene synthesis pathway in plants has been fully elucidated, the isoprene synthase from bacteria and algae is not yet characterised and should be a target for investigation in order to better assess its contribution to the global isoprene cycle.

In addition, the microbial ecology studies on isoprene degradation summarised here clearly indicate that isoprene degraders are widespread across a wide range of ecosystems, but to assess the contribution of microbes to the global isoprene biogeochemical cycle, it is essential to estimate their abundance, activity, and diversity in the environment. Cultivation-independent techniques, such as DNA-SIP and *isoA* probes, have been successfully applied to terrestrial, marine, freshwater, and phyllosphere samples to study the abundance and diversity of isoprene degraders. However, we cannot discard the possibility that other phylogenetically diverse *isoA* genes or different and novel pathways of isoprene degradation from uncultured microorganisms have been missed. To that end, the combination of DNA-SIP with other cultivation-independent techniques, such as protein-SIP [73] or Raman microspectroscopy coupled to single-cell genomics [74,75], will be key approaches in future studies to elucidate novel isoprene metabolic pathways, identify the active isoprene degraders in a microbial community, and extend the currently known diversity of isoprene-utilising bacteria. For example, protein-SIP has proven to be a successful technique not only to ratify the phylogeny of the most active microorganisms in the bacterial community revealed by DNA-SIP, but also to reconstruct complete metabolic pathways [76,77]. In addition, protein-SIP studies performed on environmental samples have allowed the identification of uncultivated bacteria and novel uncharacterised proteins involved in processes of interest, such as anaerobic methane oxidation or antibiotic degradation, which in turn can be targeted for functional biochemical studies [77,78]. Finally, protein-SIP can also inform subsequent focused proteomics studies to quantify key enzymes of metabolic processes. Such data could then be integrated into models of carbon, nitrogen, or sulfur fluxes to assess the environmental significance of a metabolic pathway of interest [77]. Similarly, Raman microspectroscopy performed with ^13^C-labelled substrates, alone or in combination with heavy water, is a very promising next-generation physiology approach to the link structure and function of a microbial community in its natural habitat [74,75,79,80]. One of its many advantages is that, as a rapid and non-destructive technique, Raman microspectroscopy can be combined with Raman-activated cell sorting (RACS) to separate isotopically labelled cells from the rest of the microbial community. In turn, sorted cells can be used for single-cell genomics or as an inoculum for cultivation, which in turn enables in-depth studies of the physiology and biochemistry of isolates [81,82,83]. However, although Raman microspectroscopy has proven to be a very powerful technique to reveal the function and interactions of complex microbial communities in their natural environments, it still faces some challenges to be broadly applied in microbial ecology studies. Firstly, the appropriate instrumentation required for Raman microspectroscopy is rather expensive, and consequently is not widely available [74,79]. Secondly, cells have to be separated from the sample matrix prior to Raman analysis, which can lead to preferential cell recovery. Therefore, the cell extraction protocol should be optimised for each specific sample to minimise the risk of preferential recovery [84]. In addition, genome amplification from single cells is commonly achieved by multiple displacement amplification, which can lead to, e.g., chimaera formation, erroneous nucleotide incorporation, or uneven genome coverage. However, most of these issues can be solved with long mate-pair libraries, high sequence coverage, and post-sequencing normalisation [75,79]. Finally, RT-qPCR assays on environmental samples using the *isoA* probes and metatranscriptomic experiments [43,85,86] will be useful approaches to elucidate who are the most active isoprene degraders in a particular environment.

There is evidence of the enrichment of particular taxa when soils are subjected to atmospherically relevant concentrations of isoprene [39,87], and the cultivation of such bacteria might allow potentially different catabolic pathways to be discovered, and thus broaden the base from which primers/probes can be refined or new ones designed. In addition, there is a culture of a homoacetogen that can use isoprene as an electron acceptor [63], and there may well be more anaerobes that can metabolise isoprene that have evaded cultivation. Undoubtedly, such anaerobes would have catabolic pathways that are different from aerobes and potentially distinct from, e.g., alkene-oxidising anaerobes. Therefore, the identification of these pathways, combined with primer/probe design, would provide a means to explore the relative contribution of anaerobic and aerobic isoprene-degrading microbes in diverse environments. 

Thus, now that an improved molecular toolbox to study the ecology of isoprene degraders is available, what is required is a systematic survey of isoprene-utilising microorganisms in contrasting environments. Of particular interest will be soils and canopies from high isoprene-emitting trees used as biofuel crops, such as willow, poplar, oil palm, or eucalyptus, since the large amounts of NOx and VOCs released by large plantations of these trees could have a detrimental effect on air quality, human health, and impede progress towards the United Nations sustainable development goals [6,16,88]. Therefore, studying the production and biological degradation of isoprene in depth in large plantations of these crops would provide data to assess the extent to which microbes might mitigate the effects of this climate-active gas in these regions. It will be also interesting to compare the abundance, diversity, and activity of isoprene degraders between high and low isoprene-emitting trees to study if and how isoprene shapes the microbial community associated with plants. Other environments that have been reported to house isoprene-producing organisms, but remain relatively unexplored, e.g., marine and freshwater environments, wetlands, or mosses [33,89,90,91], also need to be surveyed with a multidisciplinary approach, including flux measurements and molecular ecology techniques, to inform global models of isoprene cycling. In particular, production and consumption should be teased apart by using ^13^C-labelled isoprene [92]. Diurnal and seasonal isoprene flux measurements and transcript analyses will provide a more complete picture of environments as isoprene sources or sinks, and how production and consumption are influenced by environmental parameters. 

Another key aspect that requires further investigation is how isoprene metabolism is regulated, both with model isoprene degraders in the laboratory and subsequently in the environment. Although putative transcriptional regulators within the isoprene degradation gene clusters of different isoprene utilisers have been identified, the exact mechanisms by which they control isoprene degradation are not known. Targeted mutagenesis and expression studies, along with reporter strains and transcriptomic experiments will provide valuable data to identify the promoters, regulatory proteins, and environmental conditions that regulate isoprene metabolism. 

Finally, a detailed characterisation of IsoMO from representative isolates, including kinetics, affinities, substrate specificities, and the effects of inhibitors, will provide valuable information about the existence of “high affinity” and “low affinity” isoprene degraders in the environment, as is the case for other SDIMO-containing microorganisms, such as methanotrophs [93], while additionally recognising that there may be multiple pathways for isoprene oxidation. These data will also allow the comparison of the activity of IsoMO with that of related oxygenases that can co-oxidise isoprene, such as sMMO [53,94], and establish what is the contribution of these enzymes to the biological sink for isoprene.

## Figures and Tables

**Table 1:** Isoprene-degrading bacterial community from terrestrial, phyllosphere, and estuarine environments identified by DNA Stable-Isotope Probing (DNA-SIP). The dominant genus in each environmental sample incubated with ^13^C-isoprene is indicated in bold.

Study	Environment	Isoprene Concentration (ppm)	Active Isoprene Degraders
El Khawand et al., 2016 [51]	Willow soil	5000	***Rhodococcus**, Variovorax, Comamonas*
Johnston et al., 2017 [57]	Estuarine water and sediment	2000	***Microbacterium**, Rhodococcus, Mycobacterium, Gordonia*
Crombie et al., 2018 [43]	Poplar leaves	500	***Rhodococcus**, Xanthomonadaceae, Comamonadaceae*
		150	***Rhodococcus**, Variovorax*
Larke-Mejía et al., 2019 [56]	Willow soil	25	***Ramlibacter**, Variovorax, Rhodococcus*
Carrión et al., 2020 [42]	Oil palm leaves	25	***Gordonia**, Zoogloea*
	Oil palm soil	25	***Pelomonas**, Novosphingobium, Rhodoblastus, Sphingomonas*
Larke-Mejía et al., unpublished [45]	Oil palm leaves	25	***Gordonia**, Sphingomonas, Aquincola*
	Oil palm soil	25	***Aquabacterium**, Rhodococcus, Saccharibacter*
